# A Rare Presentation of Hydralazine-Induced Lupus in the Setting of Pericarditis With Concomitant Angioedema

**DOI:** 10.7759/cureus.38376

**Published:** 2023-05-01

**Authors:** Scott C Everett, Deepasri Ananth, Andrew L Alejo, Timothy Shaub, Jonathan Gan

**Affiliations:** 1 College of Medicine, Northeast Ohio Medical University, Rootstown, USA; 2 Internal Medicine, Summa Health, Akron, USA

**Keywords:** systemic lupus erythematosus, antibodies, angioedema, pericarditis, drug-induced lupus

## Abstract

Drug-induced lupus (DIL) usually presents after starting a medication known to induce DIL. However unusual presentations are rare, as such, our patient presented with initial signs and symptoms of pericarditis. Once treated as such, he progressively declined to symptoms of angioedema and worsening cardiopulmonary status. On first admission, the patient presented with chest pain that was worsened by laying down and improved by sitting up. CT Angiography (CTA) showed mild pericardial effusion, and EKG showed diffuse ST elevation, both suggestive of pericarditis, for which the patient was discharged on colchicine. The patient was readmitted one day later with swelling of the neck and tongue. The patient was re-evaluated, tested for autoantibodies, and found a positive antinuclear antibody (ANA) suggesting a diagnosis of lupus, most likely due to hydralazine.

We report a rare presentation of drug-induced lupus initially presenting with pericarditis which evolved into worsening angioedema which has not been reported in the literature thus far. Pericarditis and angioedema may be the initial presentation for a patient with drug-induced lupus. Antinuclear and anti-histone antibodies are highly sensitive and specific respectfully for drug-induced lupus. Early diagnosis and time-appropriate discontinuation of the offending agent for patients can be life-saving.

## Introduction

Drug-induced lupus (DIL) is an exogenously induced auto-immune disorder precipitated by various medications such as hydralazine (high risk), isoniazid, sulfamethoxazole-trimethoprim, procainamide, anti-tumor necrosis factor (anti-TNF) drugs (low risk) and others [1]. This condition is characterized by formation of auto-antibodies such as anti-nuclear antibodies (ANA) and anti-histone antibodies which can cause various manifestations throughout multiple organ systems.

Compared to idiopathic systemic lupus erythematosus (SLE), DIL presents more frequently in older adults. DIL most commonly presents with a combination of systemic and musculoskeletal symptoms, such as fever, weight loss, arthralgias, and serositis. However, central nervous system (CNS), renal, gastrointestinal (GI), and cutaneous findings are far more common in SLE than in DIL [2]. The dermatological findings characteristic of idiopathic SLE are fairly uncommon in DIL, however it can vary based on the offending agent responsible for the specified case of DIL [2]. Cutaneous drug-induced lupus erythema (CDILE) is a group of known skin findings associated with DIL. The most common cutaneous manifestations are bullous, erosive, or toxic epidermal necrolysis-like lesions, and often this can mimic small-vessel vasculitis with purpura and ulcerative lesions [3]. Angioedema is characterized by extravasation of fluid into the subcutaneous interstitial tissues. There are two common types: mast cell-mediated (histaminergic) and bradykinin-induced, although the specified mechanism is unclear for many drug-induced episodes of angioedema. Initial manifestations of angioedema may be insignificant, but there is potential for angioedema to progress into life-threatening compromise of the airway [4].

We report a rare presentation of drug-induced lupus in the setting of pericarditis with concomitant angioedema. There have been numerous case reports of DIL associated with pericarditis and a few case reports of patients with DIL-associated angioedema. None of these case reports had evidence of patients presenting initially with pericarditis secondary to DIL that would later evolve into angioedema. To the best of our knowledge this is the first case report with such simultaneous presentation. Informed consent has been obtained from the patient for the purpose of research and publication. There is no information directly revealing the identity of the patient. 

## Case presentation

A 49-year-old White male presented to the ED with chest pain in late 2022. This patient had a relatively complicated past medical history including type 2 diabetes mellitus (T2DM), stage 3a chronic kidney disease (CKD), hypertension, chronic anemia, and chronic heart failure with preserved ejection fraction (HFpEF). T2DM has been uncomplicated and managed with empagliflozin. Hypertension was diagnosed three years prior to this admission and has been managed with hydralazine, chlorthalidone, nifedipine, and carvedilol. Other medications for this patient include acetaminophen, aspirin, atorvastatin, and a vitamin B12 supplement. Surgical history was unremarkable except for a cardiac catheterization in 2019.

During the patient’s first admission he endorsed chest pain that began the night before. The chest pain worsened when he was supine and was relieved by sitting up. The chest pain radiated to his jaw and his left shoulder. The patient denied any nausea, vomiting, shortness of breath, or diaphoresis. The patient also denied headache, fever/chills, syncope, and hematuria, as well as any sick contacts. It is to note prior to the first ED visit, the patient had been seen in the ED for worsening respiratory status. Due to his labs showing high white blood count, arterial blood gases showing respiratory acidosis, and elevated blood urea nitrogen and creatinine, he was diagnosed with bacterial pneumonia with acute hypoxic/hypercapnic respiratory failure. Patient was subsequently intubated in the ED due to insufficient oxygenation and need for mechanical ventilation.

**Figure 1:**
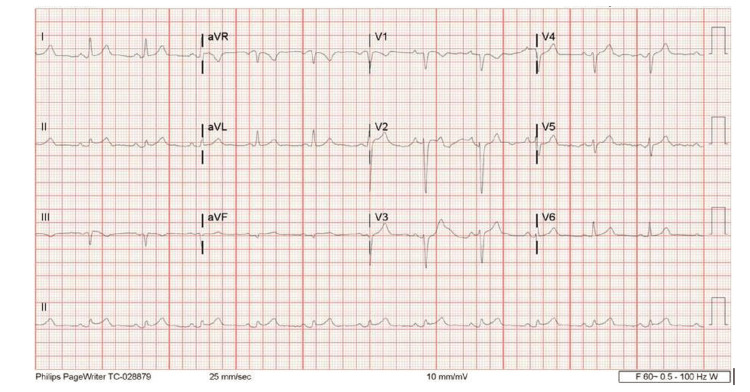
Electrocardiogram in the Emergency Department Initial EKG showed ST elevations with a concave-up morphology and concomitant PR depressions in predominantly inferior and anterior leads. This is a classic finding seen in roughly 50% of acute-phase pericarditis.

**Figure 2:**
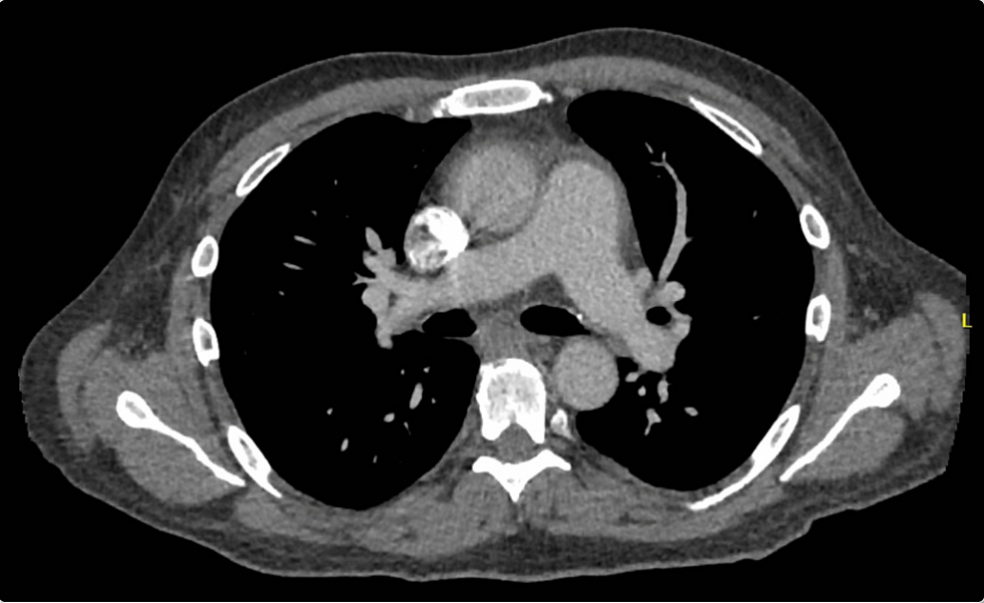
Initial CT Angiography Mild pericardial effusion surrounding the heart as seen in the increased thickness of the pericardial border with fluid-density attenuation.

**Figure 3:**
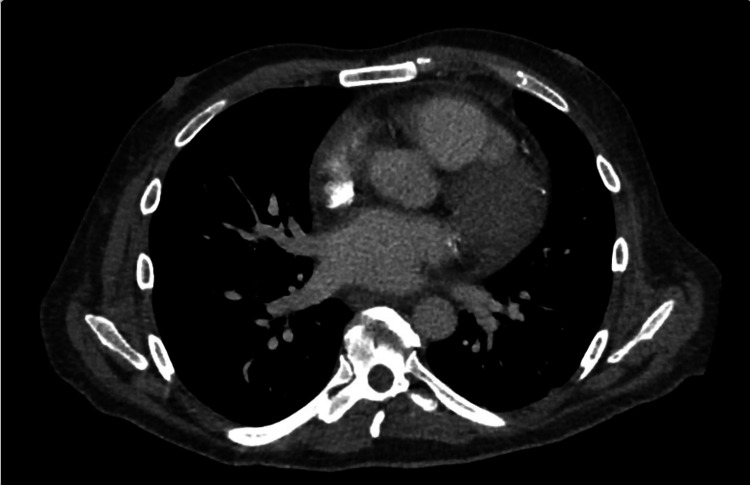
Initial CT Angiography (subsequent slice) Mild pericardial effusion surrounding the heart as seen in the increased thickness of the pericardial border with fluid-density attenuation.

Vitals at this time were stable and his physical exam demonstrated a systolic ejection murmur. Initial workup included complete blood count, complete metabolic profile, B-type natriuretic peptide (BNP), troponin, and an electrocardiogram (EKG). Pertinent positive lab findings include elevated creatinine (1.95mg/dL), BNP (1661pg/mL), and C-reactive protein (CRP; 75.1mg/L). EKG showed diffuse ST elevations and PR depressions (Figure 1). Cardiac enzyme values were within normal limits. Imaging for this patient included CT Angiography (CTA) as well as a Chest X-Ray (CXR). CTA showed mild cardiomegaly with small pericardial effusion without evidence of pulmonary embolism (Figures 2, 3). CXR showed small left effusion but was otherwise unremarkable. Evidence of pericardial effusion as well as diffuse ST elevation supported an initial diagnosis of acute pericarditis. The patient was started on colchicine. The patient was admitted to the medicine service for further evaluation. Chest pain resolved with treatment. He was discharged in stable condition from his first hospital stay the next day with scheduled follow-up with cardiology.

The patient returned to the ED that same evening due to swelling of his neck and tongue. He denied dyspnea but endorsed cough and occasional drooling. He also denied fever, weight loss, chest pain, shortness of breath, and nausea/vomiting. The patient was initially treated with a combination of epinephrine, diphenhydramine, and methylprednisolone for suspected angioedema. He was admitted to the ICU for evaluation of angioedema and possible airway compromise. Vitals were stable with a slight increase in respiratory rate (17 breaths/min). Physical exam showed normal movement of air in lungs bilaterally. Prominent swelling was noted in the submandibular and tongue regions. Swelling was Mallampati 3 and tender to palpation. The patient was started on dexamethasone. Initial workup included C1 esterase inhibitor Ag, as well as C3 and C4 levels. He was kept nothing by mouth (NPO) overnight in case a surgical procedure became necessary. On day two of admission, he endorsed improved swelling without any tenderness and normal breathing. Nephrology was consulted for CKD management, as well as workup for angioedema. Drug-induced angioedema was suspected, however in general, colchicine is much less likely to be the offending agent compared to angiotensin converting enzyme inhibitors or angiotensin receptor blockers, both of which this patient does not take. The patient had a positive ANA and high titer histone antibody as well as positive myeloperoxidase autoantibodies and proteinase-3 (Table 1). A kidney biopsy was ordered due to the presence of autoantibodies and hematuria to evaluate for antineutrophilic cytoplasmic antibody (ANCA)-associated vasculitis. He was discharged in an optimized condition. Colchicine was continued due to reduced suspicion of causing angioedema. Hydralazine was discontinued due to possible contribution to vasculitis. He was discharged on prednisone with a steroid taper and follow-up was scheduled with nephrology and rheumatology. 

**Table 1:** Antibody Titers RNP: ribonucleoprotein, SSA: Sjögren’s syndrome type A, SSB: Sjögren’s syndrome type B, MPO: myeloperoxidase, ANA: anti-nuclear antibodies

	Results	Reference
Anti-Histone	7.8	0.0-0.9 AU/mL
Anti-Sm/RNP	<0.2	<1.0 AU/mL
Anti-RNP	<0.2	<1.0 AU/mL
Anti-SSA	<0.2	<1.0 AU/mL
Anti-SSB	<0.2	<1.0 AU/mL
Anti-Chromatin	1.1	<1.0 AU/mL
Anti-MPO	168	0-19 AU/mL
Anti-Serine Proteinase 3	80	0-19 AU/mL
Anti-DNA	<1:10	<1:10 titer
ANA	> or = 1:5120	<1:80 titer

Rheumatology follow-up evaluated for autoimmune etiologies of the patient’s presentation. Based on the patient’s history and lab findings, the highest suspicion was for hydralazine-induced vasculitis or hydralazine-induced lupus. Lupus can cause the C1 esterase inhibitor deficiency that often precipitates angioedema; however, the levels of this antigen were not decreased. Prednisone was continued for this patient with a scheduled follow-up three weeks later. 

At this visit, the case was reviewed to determine the optimal course of treatment for this patient. The results of the kidney biopsy obtained during the hospital visit were also reviewed. Results were unremarkable and did not show presence of vasculitis, but evidence of active sediment on urinalysis suggested nephritis and management was indicated. The overall clinical picture of drug-induced autoimmune disorder with possible renal involvement, coupled with the improvement of symptoms upon addition of a steroid suggested a diagnosis of drug-induced lupus with possible development of lupus nephritis. The patient was started on a daily regimen of prednisone for disease management, as well as rituximab to address the nephritis. In addition, the patient was started on sulfamethoxazole-trimethoprim three times a week for Pneumocystis jiroveci pneumonia prophylaxis due to the immunosuppression associated with steroids. The patient continued regular follow up with rheumatology and nephrology to manage treatment of drug-induced lupus and nephritis. 

## Discussion

We describe a case of hydralazine-induced lupus for a patient who had been on the medication for over seven months as therapy for his HFpEF and later presented with pericarditis and angioedema. Follow-up with rheumatology led to further evaluation of his antibody status. Evaluation showed elevation of ANA, anti-histone, anti-chromatin, anti-myeloperoxidase (MPO), and anti-PR-3 antibodies and the suspected diagnosis of DIL was made. The presence of angioedema due to secondary C1-esterase deficiency from DIL has been reported in the literature. The complexity of this case is supported by the normal C1-esterase inhibitor antigen found with our patient, making the evaluation of his angioedema more difficult. To the best of our knowledge, this is the first case to be reported with drug-induced lupus causing angioedema in the absence of acquired C1-esterase deficiency. What makes this case unique is the initial pathology presenting as pericarditis. Which subsequently evolved into angioedema with no known etiology, leading to the rheumatologic work-up and diagnosis of DIL which has not been reported in the literature thus far. Most patients with DIL present with systemic symptoms and do not present with angioedema, making this diagnosis much more difficult. Not all patients with positive ANA or positive anti-histone antibody titers will present with DIL or angioedema from the same drugs. The variance in susceptibility is due to the different comorbidities and medications involving each case, but patients using medications known to cause DIL should be evaluated with antibody titers when etiology of their condition is unclear.

Upon review of multiple case reports and literature of angioedema, it is known that it can be caused by a variety of medications including angiotensin-converting enzyme (ACE) inhibitors, nonsteroidal anti-inflammatory drugs (NSAIDs), estrogens, and others. It is also known to be precipitated by other factors such as hereditary C1-esterase deficiency, mast cell disease, allergic reactions, and in some cases can be idiopathic [3-5]. Initial presentation can vary from swelling of lips, tongue, and other body parts. A more severe presentation involves the larynx and can progress to airway compromise if not managed appropriately. Physical examination displays non-gravitationally dependent, asymmetric, non-pitting edema. Initial evaluation includes basic chemistry, liver function test, CRP or erythrocyte sedimentation rate (ESR) and complement protein C4. A decrease in C4 levels is suggestive of hereditary or acquired C1 inhibitor deficiency [5]. The most common culprits of drug-induced angioedema are ACE inhibitors, although other drugs like dipeptidyl peptidase-4 (DPP-4) inhibitors have been associated as well [6]. These patients generally present with decreased levels of C1 esterase inhibitor antigen and decreased levels of C4 complement protein. Patients with drug-induced angioedema are not directly associated with elevated ANA and anti-histone antibodies which are indicative of drug-induced lupus. 

It is estimated that the incidence of DIL is between 15 to 30,000 cases annually, although the presentation of this disease can vary vastly from case to case [2]. Elevations of ANA and anti-histone are common in DIL, but elevation of MPO and proteinase 3 (PR3) antibodies could be seen with hydralazine although uncommon for initial presentation of DIL [1,2]. Hydralazine and procainamide are two of the highest risk medications and carry an odds ratio of 6.62 for causing lupus-like syndromes [2]. It is presumed that drug-induced lupus results from a drug-induced dysregulation of self-tolerance due to exposure to the causative agent over a prolonged period of time. ANA is highly sensitive for DIL and has been reported in 90-100% of cases. Most pharmacologic agents produce antibodies specific for histone proteins more specific for DIL, although new medications such as TNF inhibitors have been known to produce autoantibodies more commonly found in idiopathic SLE such as anti-dsDNA antibodies [2]. The symptoms of DIL are predominantly systemic, although some CNS, GI, and cutaneous involvement has been noted. Angioedema is not a common initial presentation of drug-induced lupus-like syndromes. Drug-induced lupus is a disease defined by remission upon discontinuation of the offending agent, although time to remission, negative antibody titers, and resolution of symptoms may vary from case to case. Discontinuation and avoidance of offending agents is the mainstay of therapy, although complete resolution of symptoms may take weeks to months. 

## Conclusions

We report a rare presentation of drug-induced lupus initially presenting with pericarditis which evolved into worsening angioedema in the absence of a known trigger for angioedema. This patient’s hydralazine-induced vasculitis/hydralazine-induced lupus was effectively treated without progression to airway compromise and the need for intubation. This case report emphasizes the need for time-sensitive analysis of ANA and other antibodies in cases where patients are taking agents known to cause DIL. Although presentations may vary significantly based on the drug in question or the patient’s other comorbidities, prompt diagnosis and discontinuation of the offending agent is essential. Thus far, the evolution of DIL from pericarditis to angioedema has not been reported in the literature. Long-term avoidance of medications known to cause DIL and follow-up with a rheumatologist is recommended for those who experience these complications. Prompt cessation of these medications can prevent progression of systemic disease and end up being life-saving for our patients.
